# Supplementary material for: MetaRibo-Seq measures translation in microbiomes
Source: Nat Commun. 2020 Jun 29;11:3268. doi: 10.1038/s41467-020-17081-z (PMC7324362; doi:10.1038/s41467-020-17081-z)
Supplement: Supplementary file 10 — Supplementary Data 7 [file 41467_2020_17081_MOESM10_ESM.zip › File2/Confidence_High_Taxonomy/57104_out.krona.html]

Javascript must be enabled to view this page.

members
magnitude
magnitudeUnassigned
count
unassigned
taxon
rank

57104\_out

282

282
2
superkingdom

282
1239
phylum

282
186801
class

order
186802
282

family
25
186806

25
1730
genus
2

SRS018817\_contig\_number\_2573SRS020869\_contig\_number\_23636


SRS015190\_contig\_number\_contig-100\_812.82355SRS050941\_contig\_number\_16749SRS104197\_contig\_number\_contig-100\_405.405
species
3
1263079

species
1
39485

SRS146764\_contig\_number\_28476

species
142586
12

SRS013098\_contig\_number\_contig-100\_276.281997SRS015782\_contig\_number\_46433SRS016018\_contig\_number\_11628SRS045004\_contig\_number\_17886SRS050299\_contig\_number\_contig-100\_1677.1677SRS097920\_contig\_number\_contig-100\_375.193155SRS1041095\_contig\_number\_contig-100\_48.42429SRS1055022\_contig\_number\_13253SRS143895\_contig\_number\_42681SRS147139\_contig\_number\_33593SRS147977\_contig\_number\_13450SRS149784\_contig\_number\_27966

7
1897003
species

SRS011405\_contig\_number\_4082SRS015369\_contig\_number\_contig-100\_2322.2322SRS016495\_contig\_number\_contig-100\_2882.97490SRS053214\_contig\_number\_contig-100\_1863.98081SRS064276\_contig\_number\_38614SRS064557\_contig\_number\_12983SRS144506\_contig\_number\_40744

254
186803
family


SRS045645\_contig\_number\_contig-100\_28914.67803SRS053356\_contig\_number\_34051SRS057717\_contig\_number\_4005SRS063127\_contig\_number\_contig-100\_16164.16165SRS063985\_contig\_number\_contig-100\_12429.52249SRS077231\_contig\_number\_contig-100\_18190.99670SRS140513\_contig\_number\_contig-100\_25368.25368
297314
7
species

246
39491
species

SRS011084\_contig\_number\_35905SRS011134\_contig\_number\_745SRS011239\_contig\_number\_27632SRS011302\_contig\_number\_30039SRS012849\_contig\_number\_31823SRS012969\_contig\_number\_18447SRS013158\_contig\_number\_837SRS013476\_contig\_number\_32331SRS013521\_contig\_number\_contig-100\_18082.18082SRS013940\_contig\_number\_12370SRS013951\_contig\_number\_39937SRS013965\_contig\_number\_30330SRS014235\_contig\_number\_36845SRS014287\_contig\_number\_13633SRS014313\_contig\_number\_5652SRS014412\_contig\_number\_15094SRS014459\_contig\_number\_29520SRS014613\_contig\_number\_1756SRS014683\_contig\_number\_16074SRS014736\_contig\_number\_8405SRS014855\_contig\_number\_17868SRS014923\_contig\_number\_3748SRS014948\_contig\_number\_14692SRS014979\_contig\_number\_35831SRS015065\_contig\_number\_6723SRS015217\_contig\_number\_29538SRS015264\_contig\_number\_contig-100\_1.118247SRS015431\_contig\_number\_54748SRS015486\_contig\_number\_contig-100\_1299.1300SRS015578\_contig\_number\_37123SRS015579\_contig\_number\_16058SRS015694\_contig\_number\_contig-100\_53.43893SRS015794\_contig\_number\_11497SRS015854\_contig\_number\_21317SRS016095\_contig\_number\_contig-100\_14060.93563SRS016203\_contig\_number\_13045SRS016335\_contig\_number\_41104SRS016897\_contig\_number\_contig-100\_19144.19145SRS016954\_contig\_number\_12907SRS016989\_contig\_number\_8968SRS017103\_contig\_number\_contig-100\_563.231434SRS017191\_contig\_number\_17587SRS017307\_contig\_number\_24362SRS017433\_contig\_number\_16846SRS017521\_contig\_number\_42341SRS017622\_contig\_number\_63SRS017701\_contig\_number\_21485SRS018313\_contig\_number\_10392SRS018427\_contig\_number\_32241SRS018623\_contig\_number\_706SRS018656\_contig\_number\_7607SRS018984\_contig\_number\_6115SRS019030\_contig\_number\_contig-100\_118.119132SRS019068\_contig\_number\_42792SRS019161\_contig\_number\_43028SRS019178\_contig\_number\_951SRS019267\_contig\_number\_2712SRS019286\_contig\_number\_4874SRS019381\_contig\_number\_9457SRS019445\_contig\_number\_32966SRS019496\_contig\_number\_9749SRS019601\_contig\_number\_24735SRS019685\_contig\_number\_contig-100\_1824.227973SRS019693\_contig\_number\_contig-100\_17043.17044SRS019968\_contig\_number\_16410SRS020394\_contig\_number\_12276SRS022071\_contig\_number\_28700SRS022137\_contig\_number\_contig-100\_73.124068SRS022524\_contig\_number\_8460SRS022713\_contig\_number\_4221SRS023346\_contig\_number\_7414SRS023526\_contig\_number\_23678SRS023715\_contig\_number\_20436SRS024075\_contig\_number\_14177SRS024132\_contig\_number\_42962SRS024331\_contig\_number\_35176SRS024492\_contig\_number\_contig-100\_905.99296SRS042284\_contig\_number\_6791SRS042628\_contig\_number\_3152SRS043001\_contig\_number\_contig-100\_10116.42331SRS043411\_contig\_number\_12584SRS043667\_contig\_number\_6042SRS043701\_contig\_number\_contig-100\_41.114684SRS043768\_contig\_number\_28539SRS043841\_contig\_number\_contig-100\_34353.34354SRS044535\_contig\_number\_24584SRS045244\_contig\_number\_7539SRS045739\_contig\_number\_8005SRS045826\_contig\_number\_24523SRS046068\_contig\_number\_contig-100\_2447.2448SRS046369\_contig\_number\_12160SRS046717\_contig\_number\_contig-100\_33.85878SRS047014\_contig\_number\_37409SRS047044\_contig\_number\_18748SRS047433\_contig\_number\_16836SRS048060\_contig\_number\_13542SRS048164\_contig\_number\_contig-100\_60.162684SRS048870\_contig\_number\_23187SRS048981\_contig\_number\_8067SRS049446\_contig\_number\_15174SRS049712\_contig\_number\_contig-100\_158.139478SRS049896\_contig\_number\_29623SRS049959\_contig\_number\_42712SRS050026\_contig\_number\_12448SRS050422\_contig\_number\_28680SRS050925\_contig\_number\_37857SRS050998\_contig\_number\_17011SRS051031\_contig\_number\_31547SRS051610\_contig\_number\_8383SRS051882\_contig\_number\_22712SRS052697\_contig\_number\_36877SRS053335\_contig\_number\_12107SRS053398\_contig\_number\_19746SRS054590\_contig\_number\_532SRS054905\_contig\_number\_22338SRS055017\_contig\_number\_contig-100\_15.208413SRS055982\_contig\_number\_10216SRS056273\_contig\_number\_21426SRS056519\_contig\_number\_18669SRS057478\_contig\_number\_8059SRS058070\_contig\_number\_31175SRS058723\_contig\_number\_8568SRS062427\_contig\_number\_10353SRS062654\_contig\_number\_14453SRS062701\_contig\_number\_5742SRS063040\_contig\_number\_21674SRS063190\_contig\_number\_14235SRS063370\_contig\_number\_12933SRS063518\_contig\_number\_17768SRS064973\_contig\_number\_contig-100\_1970.1970SRS065397\_contig\_number\_4714SRS065504\_contig\_number\_37958SRS074964\_contig\_number\_6470SRS075021\_contig\_number\_8515SRS075341\_contig\_number\_23975SRS075773\_contig\_number\_32012SRS075821\_contig\_number\_24470SRS075963\_contig\_number\_contig-100\_3877.3877SRS076804\_contig\_number\_21153SRS077127\_contig\_number\_3218SRS077194\_contig\_number\_31445SRS077294\_contig\_number\_18779SRS077335\_contig\_number\_9545SRS077454\_contig\_number\_19613SRS077502\_contig\_number\_1407SRS077552\_contig\_number\_29898SRS077641\_contig\_number\_4586SRS078176\_contig\_number\_13735SRS078665\_contig\_number\_6748SRS097958\_contig\_number\_12817SRS098061\_contig\_number\_14187SRS098073\_contig\_number\_18728SRS098514\_contig\_number\_contig-100\_161.4036SRS098571\_contig\_number\_12836SRS098644\_contig\_number\_12818SRS098717\_contig\_number\_23277SRS101376\_contig\_number\_7658SRS103987\_contig\_number\_25563SRS104084\_contig\_number\_8152SRS1041036\_contig\_number\_2185SRS1041037\_contig\_number\_17076SRS1041090\_contig\_number\_1201SRS1041091\_contig\_number\_18522SRS1041118\_contig\_number\_8244SRS1041133\_contig\_number\_23031SRS1041136\_contig\_number\_11945SRS1041142\_contig\_number\_5737SRS1041144\_contig\_number\_7565SRS1041145\_contig\_number\_38549SRS1041146\_contig\_number\_contig-100\_4560.4561SRS104311\_contig\_number\_45727SRS104327\_contig\_number\_4198SRS104400\_contig\_number\_60531SRS104485\_contig\_number\_3080SRS104636\_contig\_number\_26246SRS104975\_contig\_number\_6406SRS105153\_contig\_number\_15177SRS1054716\_contig\_number\_14720SRS1055034\_contig\_number\_9235SRS1055049\_contig\_number\_13481SRS1055056\_contig\_number\_13941SRS1055067\_contig\_number\_14480SRS1055069\_contig\_number\_16003SRS1055076\_contig\_number\_17202SRS1055083\_contig\_number\_5834SRS140492\_contig\_number\_12272SRS142503\_contig\_number\_29813SRS142599\_contig\_number\_contig-100\_5349.150726SRS142712\_contig\_number\_5462SRS142890\_contig\_number\_44898SRS143070\_contig\_number\_14656SRS143085\_contig\_number\_3248SRS143342\_contig\_number\_4455SRS143372\_contig\_number\_11226SRS143417\_contig\_number\_contig-100\_32550.32550SRS143466\_contig\_number\_31315SRS143523\_contig\_number\_26203SRS143598\_contig\_number\_19677SRS143780\_contig\_number\_25109SRS143991\_contig\_number\_30690SRS144135\_contig\_number\_contig-100\_509.205253SRS144183\_contig\_number\_42254SRS144362\_contig\_number\_11796SRS144537\_contig\_number\_37811SRS144714\_contig\_number\_29911SRS145308\_contig\_number\_contig-100\_23705.23706SRS145497\_contig\_number\_19873SRS146812\_contig\_number\_61491SRS146813\_contig\_number\_3325SRS146888\_contig\_number\_13128SRS147022\_contig\_number\_14243SRS147271\_contig\_number\_31872SRS147346\_contig\_number\_50290SRS147377\_contig\_number\_contig-100\_76.85063SRS147425\_contig\_number\_contig-100\_543.79613SRS147614\_contig\_number\_8596SRS148091\_contig\_number\_14452SRS148196\_contig\_number\_19326SRS148253\_contig\_number\_8194SRS148319\_contig\_number\_7284SRS148424\_contig\_number\_35437SRS148721\_contig\_number\_15936SRS148817\_contig\_number\_contig-100\_1796.41494SRS148874\_contig\_number\_6310SRS149075\_contig\_number\_contig-100\_10.124507SRS149181\_contig\_number\_3496SRS149244\_contig\_number\_7257SRS149325\_contig\_number\_14668SRS149879\_contig\_number\_22660SRS893170\_contig\_number\_11615SRS893230\_contig\_number\_8000SRS893231\_contig\_number\_3486SRS893252\_contig\_number\_20125SRS893270\_contig\_number\_20186SRS893279\_contig\_number\_17917SRS893288\_contig\_number\_4543SRS893295\_contig\_number\_8700SRS893327\_contig\_number\_9470SRS893341\_contig\_number\_contig-100\_62.93188SRS893342\_contig\_number\_5902SRS893369\_contig\_number\_4961SRS893373\_contig\_number\_24424SRS893378\_contig\_number\_23839SRS893380\_contig\_number\_518SRS971275\_contig\_number\_46089SRS971276\_contig\_number\_36919

genus
841
1


SRS015663\_contig\_number\_3128
1
2049040
species

family
2
541000

genus
1263
1

1
46228
species

SRS147766\_contig\_number\_6221

genus
1
1924093

species
1944639
1

SRS097889\_contig\_number\_14901

1
990719
family


SRS1041129\_contig\_number\_8734
species
1
1930012
